# *Micrasterias* as a Model System in Plant Cell Biology

**DOI:** 10.3389/fpls.2016.00999

**Published:** 2016-07-12

**Authors:** Ursula Lütz-Meindl

**Affiliations:** Plant Physiology Division, Cell Biology Department, University of SalzburgSalzburg, Austria

**Keywords:** autophagy, cell shaping, cell wall formation, cytoskeleton, organelle degradation, stress, TEM, EM tomography

## Abstract

The unicellular freshwater alga *Micrasterias denticulata* is an exceptional organism due to its complex star-shaped, highly symmetric morphology and has thus attracted the interest of researchers for many decades. As a member of the Streptophyta, *Micrasterias* is not only genetically closely related to higher land plants but shares common features with them in many physiological and cell biological aspects. These facts, together with its considerable cell size of about 200 μm, its modest cultivation conditions and the uncomplicated accessibility particularly to any microscopic techniques, make *Micrasterias* a very well suited cell biological plant model system. The review focuses particularly on cell wall formation and composition, dictyosomal structure and function, cytoskeleton control of growth and morphogenesis as well as on ionic regulation and signal transduction. It has been also shown in the recent years that *Micrasterias* is a highly sensitive indicator for environmental stress impact such as heavy metals, high salinity, oxidative stress or starvation. Stress induced organelle degradation, autophagy, adaption and detoxification mechanisms have moved in the center of interest and have been investigated with modern microscopic techniques such as 3-D- and analytical electron microscopy as well as with biochemical, physiological and molecular approaches. This review is intended to summarize and discuss the most important results obtained in *Micrasterias* in the last 20 years and to compare the results to similar processes in higher plant cells.

## Introduction

**FIGURE 1:**
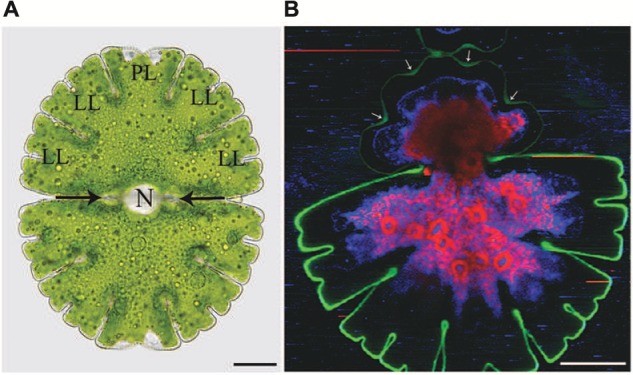
**Light microscopic **(A)** and Raman spectroscopic **(B)** image of *Micrasterias denticulata.* (A)** The cell consists of two semicells that are connected by an isthmus (black arrows). Each semicell has one polar lobe (PL) and four denticulated lateral lobes (LL). The nucleus (N) is located in the cell center. **(B)** The different colors of the Raman image represent chemically different regions identified by non-negative matrix factorization. The green color represents the cellulosic cell wall which is more distinct and thicker in the non-growing old semicell, when compared to the newly formed young semicell (upper part). In the young growing semicell, the cell wall in the area of the indentations is highlighted more intensively (arrows) than at the lobe tips. Raman spectroscopic image kindly provided by Notburga Gierlinger.

Among the placoderm desmids the genus *Micrasterias* has an exceptional position due to its highly ornamented, star-shaped morphology with deep indentations and furcated lobe tips (**Figure 1A**). By their beauty, their high symmetry and their flat, disk-shaped cell architecture facilitating any microscopic analysis as well as their close relationship to higher plants (Wodniok et al., 2011; Leliaert et al., 2012) *Micrasterias* cells have lent themselves as excellent model systems for studying plant cell morphogenesis. In many aspects results obtained in *Micrasterias* cells are applicable to higher plants and comparison with them additionally provides information on the evolution of cellular processes.

Early investigations around the turn of the 19th century have already focused on cell shape formation of this extraordinary organism (Hauptfleisch, 1888; Lütkemüller, 1902) and the implementation of an appropriate nutrient solution for their easy cultivation (Pringsheim, 1930; Waris, 1950a) represented the basis for numerous further studies. Whereas the very early investigations were intended to find an inner “cytoplasmic framework” for the morphology of *Micrasterias* (Waris, 1950b) subsequent studies focused on the peripheral cytoplasm (Teiling, 1950) and the nucleus (Waris and Kallio, 1964) as shape determining units. At a time where genetic control of cellular processes was far from being understood these studies (Kallio, 1949; Kallio and Heikkilä, 1972; Kallio and Lehtonen, 1981) provided interesting insight into cytopmorphogenesis by showing that a three-lobed pre-stage of a young semicell of *Micrasterias* can be formed even when the nucleus is physically removed. Further differentiation into lobe tips and indentations, however, requires continuous nuclear control. An increase in ploidy increases the complexity of the cell pattern and leads to triradiate or quadriradiate *Micrasterias* cells (for summary see Kallio and Lehtonen, 1981).

Kiermayer (1964) who tested several *Micrasterias* species for their suitability as cell biological model system in respect to growth and reproduction properties and their sensitivity to experimental and environmental impact, was the one who selected the species *Micrasterias denticulata* and defined its developmental stages in 15 min intervals. This represented the basis for his first investigations on ultrastructural details during morphogenesis (Kiermayer, 1968, 1970a) and for numerous other studies on cell physiology, cell wall formation, secretion, cytoskeleton function, and environmental impact in *M. denticulata* in the last decades (for references see below).

The most important insights into cytomorphogenesis arising from Kiermayer’s studies and summarized by Kiermayer (1981), Kiermayer and Meindl (1984), and Meindl (1993) were that the large dictyosomes of a *Micrasterias* cell consist of a constant number of 11 cisternae throughout the cell cycle and that they switch a several times during morphogenesis to form the different vesicle populations that contain cell wall precursors for septum-, primary- and secondary wall formation. These results obtained by standard chemical fixation were confirmed in a later study on high pressure frozen *Micrasterias* cells (Meindl et al., 1992). The contents of the different vesicle populations observed by Kiermayer were defined by immuno-transmission electron microscopy (TEM) and immunofluorescence experiments in the confocal laser scanning microscope (CLSM) using antibodies against cell wall constituents such as, pectins, different hemicelluloses and arabinogalactane proteins (AGP; Lütz-Meindl and Brosch-Salomon, 2000; Eder and Lütz-Meindl, 2008; Eder et al., 2008 see also below). Additionally, by simple turgor reduction experiments Kiermayer’s studies (Kiermayer, 1964, 1967, 1981) demonstrated impressively that the plasma membrane contains a pre-pattern for morphogenesis in form of “membrane recognition areas” for the cell wall delivering vesicles and thus plays the mayor role in cell shaping of *Micrasterias.* The “membrane recognition areas” postulated by Kiermayer were later shown to be zones of continuously changing, local calcium influx at the respective lope tips during development of the cell pattern (Meindl, 1982a,b; Troxell et al., 1986; Troxell, 1989; Troxell and Scheffey, 1991). They correspond to the fusion sites of primary wall material delivering Golgi vesicles as shown by means of autoradiography (Lacalli, 1975) and by TEM in high pressure frozen developmental stages (Meindl et al., 1992).

Dobberstein and Kiermayer (1972) were the first who described hexagonally ordered “rosette” complexes inside of a particular Golgi vesicle population, the flat vesicles, which are delivered to the plasma membrane, where they are responsible for formation of cellulose fibrils. *M. denticulata* was thus the first plant cell in which cellulose formation was discovered. By Kimura et al. (1999) it was proven in vascular plants by means of immuno labeling and freeze fracture technique in TEM that the rosette terminal complexes are the sites of cellulose formation. In *Micrasterias* an endogenous cellulose synthase was localized many years later by means of transient genetic transformation (Vannerum et al., 2010).

Another finding that goes back to Kiermayer and gives information on the basics of growth and morphogenesis in *Micrasterias* is the perception that continuous protein synthesis is required throughout its morphogenesis and that any experimental interruption by employment of different RNA or protein synthesis inhibitors is reflected in characteristic cellular reactions summarized as “anuclear type of development” (ATD) syndrome (Kiermayer and Meindl, 1980, 1986). Its typical morphological appearance is a reduction of the cell pattern to three to five cylindrical lobes which lack further differentiation. As such shape malformations have so far only been found in *Micrasterias* cells that grow without nuclear control (summarized by Kallio and Lehtonen, 1981) and are not inducible by any of the numerous drugs and inhibitors that have been tested on *Micrasterias* the term “ATD” syndrome was invented for it. Besides the characteristic cell shape malformation the ATD syndrome comprises prolonged extension of the primary wall from 5 h in controls up to 24 h. Consequently the cells burst due to a lack of secondary wall formation. Additionally the structure of the primary wall is altered and the number of dictyosomes is reduced. Knowledge on the characteristics of this syndrome allows easy detection of any impairment of protein biosynthesis in *Micrasterias*, e.g., by experimental or environmental influence.

Kiermayer’s, (1968) excellent TEM images on microtubule distribution around the nucleus in growing and non-growing *Micrasterias* cells also inspired further research on the involvement of the cytoskeleton in intracellular organelle migration. During growth the large nucleus moves from its position in the central constriction of a *Micrasterias* cell, called isthmus (**Figure 1A**), into the expanding semicell and returns to the cell center at the end of morphogenesis. Coincidently the chloroplast which has duplicated before mitosis, expands itself into the growing semicell. Its shape copies the outer cell shape at the end of development. Both organelle movements are cytoskeleton dependent (Kiermayer, 1970b; Meindl and Kiermayer, 1982; Meindl, 1983, 1992; for summary see Meindl, 1993; Lütz-Meindl and Menzel, 2000). During its migration the nucleus is surrounded by a basket-shaped microtubule system that also contains actin filaments (Meindl et al., 1994). It originates from a distinct microtubule center and stays in contact with the cell center by a continuously elongating microtubule band during nuclear migration into the growing semicell. Along this band the nucleus moves back into the isthmus where it is anchored by a ring shaped microtubule band after morphogenesis. This band corresponds to the pre-prophase band of higher plant cells (Pickett-Heaps and Northcote, 1966; Karahara et al., 2009). Any physical or chemical disruption of microtubules during growth of *Micrasterias* leads to an abnormal dislocation of the nucleus into the cell periphery and prevents further cell divisions (Meindl, 1983; Holzinger et al., 2002). Several studies indicated that the actin binding protein profilin, myosins and kinesis-like proteins are involved in regulation and/or achievement of nuclear and chloroplast migration in growing *Micrasterias* cells (Holzinger et al., 2000; Holzinger and Lütz-Meindl, 2002; Oertel et al., 2003). As the nucleus of *M. denticulata* measures about 30 μm in diameter and is thus easily detectable even with a dissecting microscope, the alga is a perfect system for identifying activity of anti-microtubule drugs in a plant cell by a simple methodological approach (Meindl and Kiermayer, 1981). Any dislocation of the nucleus indicates a dysfunction of the microtubule system.

Whereas cell shaping and intracellular regulators of growth and cytomorphogenesis have been in the center of research on *Micrasterias* for several decades, the suitability of this alga as sensitive model for studying environmental impact has gained importance in the recent years. *Micrasterias* and other members of the family Desmidiaceae mostly inhabit acid peat bogs all over the world, from tropic climates to Polar Regions and from sea level up to more than 3000 m altitudes (see also Brook, 1981). Their natural habitats are shallow bog ponds that may be exposed to rapidly changing environmental conditions and may face extreme parameters such as intense sun light, high UV irradiation, drought, increasing salinity, very low, but also high temperature as well as impact of man-made pollutants such as heavy metals, pesticides or fertilizers.

Based on earlier results briefly outlined in the Section “Introduction” and on a more detailed survey on results obtained in the last 20 years, this review aims to provide an overview on our present knowledge on the cell biological basis for growth and cell shape formation as well as on responses of *Micrasterias* to different abiotic stress scenarios. Recent results on dictyosomal function, cell wall composition, the cytoskeletal and ionic regulation of growth, stress-induced organelle degradation, occurrence and induction of autophagy and programmed cell death as well as on accompanying physiological parameters will be in the focus of this review. Comparison to similar processes in closely related higher land plants will be drawn in each chapter and an outlook on future research topics on *Micrasterias* will complete this work.

## Cell Biological Basis of Growth and Shape Formation

### Cell Wall Development and Function of Dictyosomes

Cell development and pattern formation of *M. denticulata* (summarized by Meindl, 1993) starts with the formation of a thin, fragile septum wall at the overlapping zone of the two parental secondary cell walls in the isthmus during late mitosis. The two halves of a *Micrasterias* cell are separated when the septum closes like a diaphragm. The septum consists mainly of high-methyl esterfied pectins that are delivered to the growing septum by a particular “septum vesicle” population as shown by immunogold labeling with JIM antibodies (Lütz-Meindl and Brosch-Salomon, 2000). Septum formation lasts about 15 min and is followed by the process of primary wall formation and shaping. According to our present knowledge shaping of *Micrasterias* cells is regarded as a temporal and spatial sequence of repeated growth cessation at particular, symmetrically arranged points at the cell periphery. While the first bulge that develops from the parental semicells grows uniformly in the main plane of the cell, cessation of growth at two symmetrical points after about 75 min leads to the formation of the first two indentations that develop to the deepest at the final cell shape. They define the species specific morphology of a *M. denticulata* cell. Subsequent formation of the residual indentations follows the same principle. In the areas between the growth cessation points, deposition of primary wall material and expansion is continued. Growing and non-growing zones alternate during shape formation and the number of both increases with proceeding development. In early stages the single lobes perform tip growth which from its course resembles tip growth of pollen tubes, root hairs or moss protonemata (Vidali and Bezanilla, 2012; Gu and Nielsen, 2013; Rounds and Bezanilla, 2013). However, several tips of a *Micrasterias* cell grow at the same time. This process has been defined as “multipolar tip growth” (Kiermayer and Meindl, 1989; Lütz-Meindl and Menzel, 2000), an unique phenomenon that represents an enormous demand for a single cell. Finally the outer cell shape of *M. denticulata* is completed about 5 h after mitosis by furcation of the lobe tips. Underneath the primary wall a thick cellulose rich secondary wall is deposited that contains pores. The primary wall is finally pushed off by a sudden onset of mucilage production through the secondary wall and its pores (for details see Oertel et al., 2004).

**FIGURE 2:**
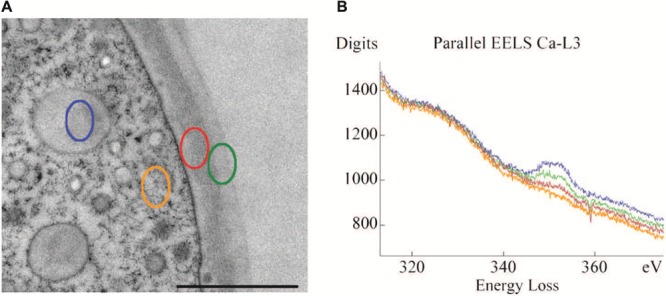
**Transmission electron microscopy (TEM) micrograph **(A)** of cell periphery and primary cell wall of *M. denticulata* with highlighted measurement sites of EEL spectra (scale bar is 1 μm). (B)** EEL spectra acquired at the Ca L_2,3_-edge, indicating calcium binding capacities of different cell components after doping with calcium acetate. Outer side of primary wall (green) has higher calcium binding capacity than inner side (red). Mucilage vesicle (blue) reveal highest calcium binding capacity, the cytoplasm (orange) lowest. Reprinted with permission from Eder and Lütz-Meindl (2008), Copyright^©^ 2008 Royal Microscopical Society.

Achievement of the complex cell pattern of a *Micrasterias* cell requires particular physical properties of the primary cell wall that allow intussusception of wall material on the one hand and stiffening and growth arrest on the other hand. Recent Raman spectroscopic investigations (**Figure 1B**) clearly show that the cellulose content of the primary wall of the outgrowing lobes is much lower than that of the indentations in which the cell wall is thicker and less extensible. This technique also demonstrates unambiguously the considerable difference in thickness and cellulose content between the growing primary and the non-growing secondary cell wall. The degree of pectin esterification which determines the calcium-binding capacity accounts for the flexibility of the cell wall during primary wall growth. A combination of immuno TEM, immuno fluorescence, de-esterification studies and calcium measurements by TEM-coupled electron energy loss spectroscopy (EELS) has shown (Lütz-Meindl and Brosch-Salomon, 2000; Eder and Lütz-Meindl, 2008) that pectic polysaccharides are transported to the cell wall in a de-esterified form inside of a particular Golgi vesicle population (D-vescicles according to Kiermayer, 1970a,b; Meindl et al., 1992; Lütz-Meindl and Brosch-Salomon, 2000) and become methyl-esterified at the inner side of the developing primary wall (Eder and Lütz-Meindl, 2008). This allows flexibility and integration of new wall material. While they are translocated toward the outer side of the wall they become again de-esterified (see also Lütz-Meindl and Brosch-Salomon, 2000) and are thus able to bind high amounts of calcium (**Figures 2A,B**) which leads to wall stiffening and growth cessation. Enzyme activity assays provided evidence for the existence of a pectin-desterifying enzyme in *M. denticulata*. Moreover, enhanced desterification by experimental addition of a pectin methylesterase that de-esterifies pectins in higher plants, resulted in growth inhibition and shape malformation of *Micrasterias* (Eder and Lütz-Meindl, 2008). This indicates that as in higher plant cells (Carpita and Gibeaut, 1993; Goldberg et al., 2001; Bosch et al., 2005; Bosch and Hepler, 2005) the de-esterification process and its regulation is crucial for morphogenesis and growth of *Micrasterias* (Eder and Lütz-Meindl, 2008). Though it is likely, there is so far no indication that the degree of esterification is different in the areas of the forming indentations in comparison to the zones of the outgrowing lobes.

The existence of pectins and particularly of homogal-acturonans as dominant polysaccharide in growing cell walls has been also demonstrated in other members of Desmidiaceae such as *Closterium* and *Penium* (Baylson et al., 2001; Domozych et al., 2007) as well as in other green algae (Popper and Fry, 2003). By extraction of isolated cell walls of *Penium* a particular homogalacturonan was identified that was partially esterified and was equivalent to that of land plants. As in *Micrasterias*, the cell wall of *Penium* was recognized by the antibodies JIM 5 and JIM7 and the degree of de-esterification increased with the distance from the isthmus region (Domozych et al., 2006; Domozych et al., 2007). The degree of methyl-esterification in the growing primary wall of *Micrasterias* corresponds well to findings in higher plants (Bush and McCann, 1999). However, the detailed structure of pectins in desmids is not yet known. There are some indications that algae contain higher contents of galacturonic and glucuronic acid than higher plants (Popper and Fry, 2003).

Mucilage vesicles of *Micrasterias* reveal the highest calcium-binding capacity among all cytoplasmic components measured by EELS indicating a high amount of low-methyl-esterified pectins in the mucilage during its transport to the cell periphery (**Figures 2A,B**; Eder and Lütz-Meindl, 2008). As soon as they are excreted through the cell wall or the pores (Oertel et al., 2004) their calcium-binding capacity diminishes either by enzymatic activity or by changing pH (Eder and Lütz-Meindl, 2008). This allows uptake of water and swelling, which is responsible for generating the force for directed movement of desmids with respect to light (Domozych and Rogers-Domozych, 1993) but also for protecting the cells against unfavorable environmental conditions (Oertel et al., 2004).

Additional components of the primary cell wall of *Micrasterias* that may be involved in regulation and achievement of growth and morphogenesis are AGPs and hemicelluloses. AGPs are hydroxyproline-rich proteoglycans that have been identified in the plasma membrane and in plant cell walls (Serpe and Nothnagel, 1999) and are involved in growth, development and differentiation of higher plant cells (Knox et al., 1989; Majewska-Sawka and Nothnagel, 2000; Seifert and Roberts, 2007). In *Micrasterias* AGPs have been located in the primary cell wall, in D-vesicles, in parts of the dictyosomes and along the plasma membrane of the non-growing semicell by means of antibodies specific to higher plant AGPs (Lütz-Meindl and Brosch-Salomon, 2000; Eder et al., 2008). Their presence exclusively along the plasma membrane of the non-growing semicell may indicate a regulatory role in growth. As cell wall material containing vesicles are produced at dictyosomes all over the cell and are also transported to the periphery of the non-growing semicell, a barrier function of AGPs as postulated by Kreuger and van Holst (1996) may prevent their fusion in the non-growing part of a *Micrasterias* cell.

**FIGURE 3:**
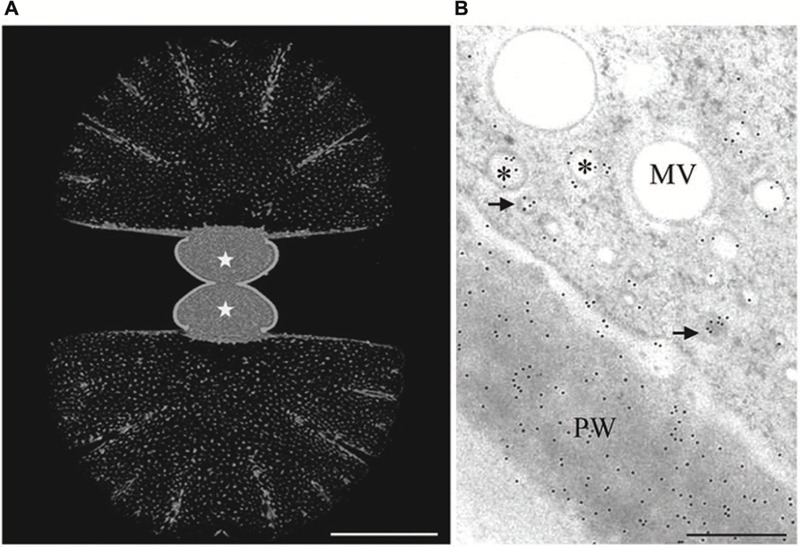
**Immuno labeling of growing *Micrasterias* cells by monoclonal anti-AGP antibody JIM13. (A)** Labeling of the primary wall of both growing semicells (asterisks) visualized in CLSM. The non-growing secondary walls of the old semicells (upper and lower part are not labeled). Scale bar is 50 μm. **(B)** TEM micrograph showing labeling of primary wall (PW) and two different vesicle populations indicated by asterisks and arrows (scale bar is 0.5 μm). Reprinted with permission from Eder et al. (2008), Copyright^©^ 2008 Phycological Society of America.

The labeling pattern of AGPs by the antibodies JIM8, JIM13 and JIM14 both by immunofluorescence in CLSM and by immunogold labeling in TEM (**Figures 3A,B**) in the primary cell wall suggests an involvement of the recognized AGP type 2 proteoglucans (see Knox et al., 1991; Yates et al., 1996) in cell development of *Micrasterias* (Eder et al., 2008). However, their distribution did not correlate with the cell pattern. The properties of the epitopes recognized at immunodot- and Western blots suggest a similar molecular weight of the AGPs in *Micrasterias* as those of higher plants (Eder et al., 2008). They are rich in galactose and xylose but in contrast to higher plants do not bind to a synthetic glycoside, the β-GlcY reagent (Yariv et al., 1962, 1967) which is generally used for their identification.

**FIGURE 4:**
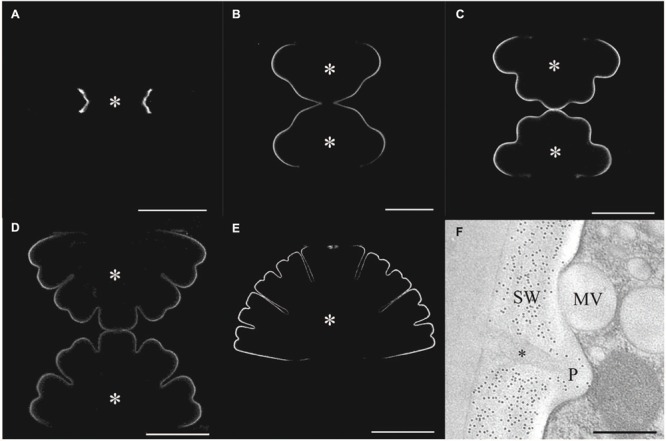
**Immuno labeling of the cell wall of developing stages of *Micrasterias*. (A–E)** Primary wall of different developing stages of *Micrasterias* labeled by polyclonal anti-xyloglucan antibody. Asterisks indicate growing semicells (scale bars are 50 μm). **(F)** Immunogold labeling of the non-growing secondary wall by BG1 antibody recognizing (1-3, 1-4)-ß-D-glucans. Mucilage (asterisk) in cell wall pore (P) and mucilage vesicle are not labeled (scale bar is 0.5 μm). Reprinted with permission from Eder et al. (2008), Copyright^©^ 2008 Phycological Society of America.

The growing primary wall of *Micrasterias* also contains xyloglucans (Eder et al., 2008) similar to those of higher land plants as indicated by fluorescence labeling (**Figures 4A–E**) by an antibody directed against higher plant epitopes (Lynch and Staehelin, 1992). Immuno TEM experiments showed that some of these epitopes are also present in the secondary wall. Binding of this antibody at the *trans*-side and in primary wall material containing vesicles (D-vesicles) suggests that these xyloglucans are secreted in *Micrasterias* similar to higher plant cells. Interestingly the secondary wall of *Micrasterias* (**Figure 4F**) but not the primary wall also contains mixed-linked glucans [(1-3, 1-4)-ß-D-glucans; Eder et al., 2008] that belong to the mayor polysaccharide component of the cell wall of grasses (Gibeaut and Carpita, 1993; Meikle et al., 1994; Trethewey et al., 2005). Similar glucans have also been identified in the alga *Ulva lactuca* and in the liverwort *Lophocolea bidentata* by (1-3, 1-4)-ß-D-glucanase digestion (Popper and Fry, 2003).

Analyses of genome-wide transcript expression of synchronized cultures with high percentages of growing cells of *M. denticulata* provided evidence for a role of Rab and SNARE cycles in vesicle fusions and for AGP-like proteins in growth and cell pattern formation (Vannerum et al., 2011). Additionally the xyloglucan-modifying enzymes xyloglucan endotransglycosylase/hydrolase (XET/XTH), class-III-peroxidases and expansins have been identified as growth and cell shape formation relevant constituents among the 107 genes identified. Phylogenetic analysis suggested that four of the identified genes showed high similarity to the expansin A family of higher plants, although their domain organization was divergent. Overexpression of one of these genes (MdEXP2) resulted in cell shape aberrations. Unfortunately so far only transient transformation is possible in *Micrasterias* (Vannerum et al., 2010*)*. A recent study on the closely related alga *Penium margaritaceum*, however, reporting on a successful stable transformation and reverse genetic analysis (Sorensen et al., 2014) are encouraging also for future studies in *Micrasterias* in this respect.

All these results show that similar to recent results in *Penium* (Domozych et al., 2014) the cell wall of *Micrasterias* generally corresponds to that of higher land plants. However it differs in details, which is of evolutionary interest as these differences may have been crucial for the colonization of terrestrial habitats. For further discussion and additional aspects on the evolution of cell walls and on terrestrialization of Streptophyta see the reviews by Graham et al. (2000), Sorensen et al. (2010, 2012), Domozych et al. (2012), Delwiche and Cooper (2015), Harholt et al. (2016).

Formation of the patterned cell wall in *Micrasterias* requires temporally orchestrated production and precise deposition of cell wall material during development. Timely supply with cell wall material is achieved by highly regulated and synchronized switching of the dictyosomes for producing vesicle populations with different contents as shown by several earlier investigations (for literature see Introduction). Thus precise definition of a developmental stage of *Micrasterias* is easily possible by a TEM image of just a few Golgi bodies and their associated vesicles. As mucilage vesicles are delivered from the dictyosomes consistently, also in non-growing cells (Oertel et al., 2004) dictyosomal activity in *Micrasterias* is maintained continuously during the cell’s life cycle. This is contrary to most other plant cells were dictyosomal secretion ceases when growth is completed.

Various cell physiological studies using inhibitors that target different steps of product formation gave insight into the reaction of the secretory machinery and have revealed similarities to higher plant cells. Disturbance of N-or O-linked glycosylations in dictyosomes by tunicamycin and brefeldin A had drastic consequences on Golgi morphology and secretion (Höftberger et al., 1995; Salomon and Meindl, 1996). As in higher plant cells (Satiat-Jeunemaitre et al., 1996) brefeldin A leading to dissociation of COP1 proteins from Golgi membranes (Nebenführ et al., 2002) resulted in a reversible reduction in the number of dictyosomes in favor of the ER in *Micrasterias* (Salomon and Meindl, 1996). In a similar way as thapsigargin (see below), the Ca-ATPase inhibitor cyclopiazonic acid prevented product supply from the ER leading to dilatations of ER cisternae and a reduction on the number of dictyosomal *cis*-cisternae (Höftberger et al., 1995) indicating the importance of calcium for proper secretion. Experimental release of nitric oxid (NO) by donors such as S-nitroso-*N*-acetyl-_DL_-penicillamine (SNAP) or sodium nitroprusside (SNP) selectively suppressed secondary wall formation in *Micrasterias* (Lehner et al., 2009) and impaired dictyosomal function probably via inhibition of enzymes such as glyceraldehyde-3-phosphate-dehydrogenase (GAPDH).

**FIGURE 5:**
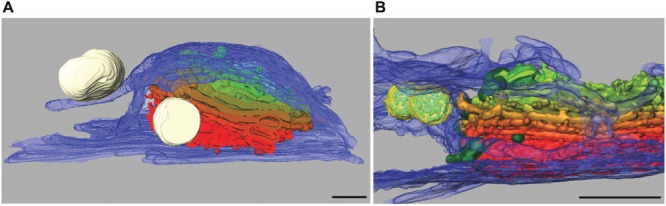
**3-D reconstruction of *Micrasterias* dictyosomes from FIB-SEM series.** *Cis*-side in red, *trans-*side in green. **(A)** The entire dictyosome is enwrapped by ER envelope (blue) (scale bar is 1 μm). **(B)** Detail of another dictyosome showing the close spatial interaction between dictyosomal cisternae and the ER. Mucilage vesicles in white, multivesicular bodies in yellow (scale bar is 1 μm). Reprinted with permission from Lütz-Meindl et al. (2015), **(A)** Copyright^©^ 2015 Royal Microscopical Society and from Wanner et al. (2013) **(B)**, Copyright^©^ 2013 Elsevier Inc.

Dictyosomes of *Micrasterias* are unique organelles measuring 2–3 μm in diameter and revealing a constant number of 11 cisternae independent from the stage of the cell cycle. They are thus many times larger than in higher plant cells or in other algae (see also Lütz-Meindl et al., 2015). In growing cells they are located in great number around the nucleus and along the chloroplast membrane. Like in higher plant cells (see e.g., Hawes and Satiat-Jeunemaitre, 2005) they have been shown to be associated with a *cis*-located ER cisternae in TEM. A recent study using focused ion beam milling and block face imaging by field emission scanning electron microscopy (FIB-SEM) has yielded first information on the 3-D architecture of Golgi stacks in *M. denticulata* (Wanner et al., 2013). This method allows 3-D reconstruction of large cytoplasmic volumes up to several hundred μm^3^ by 5–10 nm serial slicing. FIB-SEM series and 3-D reconstruction of high pressure frozen and cryo-substituted *Micrasterias* cells showed that the dictyosomes are not only associated with a *cis*-ER cisternae but are surrounded by a huge *trans*-side located ER sheath leading to an almost entire ER envelope around them (**Figures 5A,B**).

Membranes of *trans*-Golgi cisternae were found to be in contact with these *trans*-ER cisternae. This finding is particularly interesting as “*trans*-ER” systems are well known from mammalian cells (for review see Mogelsvang et al., 2004) but not from higher plant cells. It opens new insight into functional interactions between the ER and the Golgi system. Additionally this study indicates that multivesicular bodies regarded as components of the endocytic pathway are in close spatial contact not only with membranes of *trans*-Golgi compartments as postulated in higher plants (see for example Otegui and Spitzer, 2008; Robinson et al., 2008) but also with the *trans*-ER. This may indicate that both membrane systems participate in their formation in *Micrasterias*. Interconnections between dictyosomal cisternae were visualized by FIB-SEM tomography and it was shown that the Golgi stacks consist of flat unfenestrated cisternae with slightly lacerated rims (Wanner et al., 2013).

In summary this section shows that timely coordinated function of the exceptionally large and highly organized dictyosomes of a *M. denticulata* cell, in tight cooperation with its surrounding ER envelope represents a basis for morphogenesis. The products they deliver into the growing primary wall correspond essentially to that of higher plants.

### Involvement of the Cytoskeleton in Growth and Morphogenesis

The multi-lobed symmetric morphology of a *Micrasterias* cell may suggest the existence of internal fibrillary axes that provide the basis for shape generation. Early investigators postulated a “cytoplasmic framework” (Waris, 1950b) that has never been verified. Although randomly oriented microtubules have been reported in the cortical cytoplasm of growing *M. denticulata* (Kiermayer, 1968) and *M. pinnatifida* cells by TEM, it has soon turned out that chemical destruction of the these microtubules neither influences growth and cell pattern formation (for summary see Kiermayer, 1981; Meindl, 1993; Lütz-Meindl and Menzel, 2000) nor alters the patterned distribution of cellulose microfibrils in the secondary wall (Schmid and Meindl, 1992). This is contrasting to findings in numerous higher plant cells (e.g., Lloyd and Chan, 2008) and also to results in the closely related desmids *Closterium* (Hogetsu, 1992) and *Penium* (Domozych et al., 2014) both growing at one distinct tip only. It is supposed that the cortical microtubules in *Micrasterias* only represent a cytoplasmic reinforcement of the cell wall (Schmid and Meindl, 1992), while the more centrally located microtubules participate in chloroplast expansion during growth (Meindl and Kiermayer, 1982).

The actin system of *M. denticulata* was visualized by means of microinjection of fluorescently labeled phalloidin into growing cells (Meindl et al., 1994), as well as by phalloidin staining of glutaraldehyde/formaldehyde fixed unembedded cells or by actin-antibody labeling of methyl-methacrylate embedded cells (Pflügl-Haill et al., 2000). While only single actin filaments are present during early developmental stages, a distinct, dense actin filament network extending from the chloroplast surface toward the plasma membrane pervades the growing semicells as soon as cell differentiation starts. The actin cables of this network show high dynamics when labeled with fluorescent phalloidin, but no preferential cell pattern related orientation in growing cells (Meindl et al., 1994). Involvement of the actin cytoskeleton in growth and cell pattern formation in *Micrasterias* is indicated by numerous results. Primary wall material containing D-vesicles are lined up in front of their fusion sites at the lobe tips in high pressure frozen developmental stages indicating a regulatory role of filamentous structures (Meindl et al., 1992). Disturbance of the balance between filamentous actin and its globular subunits (G-Actin) by experimental injection of small amounts of the G-actin-binding protein profilin leads to retardation of growth (Holzinger et al., 1997). Immuno TEM and immuno blot studies have provided evidence for the presence of the actin-binding protein spectrin in the desmids *M. denticulata, Closterium lunula* and *Euastrum oblongum* (Holzinger et al., 1999). Additionally spectrins are known to cross-link actin filaments and to accomplish F-actin membrane interactions (for references see Holzinger et al., 1999). In growing *M. denticulata* cells this regulatory protein was found at membranes of different secretory vesicle populations and also on membranes of primary wall material containing D-vesicles. This indicates an involvement of spectrin in actin dependent vesicle transport representing the basis for growth.

Several studies in *M. denticulata* but also in other desmids have clearly indicated that any disruption of the actin filament system causes dose-dependent growth inhibition and severe cell shape malformations up to a complete loss of cell symmetry (summarized in Lütz-Meindl and Menzel, 2000). Coincidently a breakdown of the actin network in *M. denticulata* has been visualized in CLSM as consequence of incubation with the actin disorganizing drugs latrunculin B and cytochalasin D. Only clusters of non-filamentous actin and short actin filaments remained visible after this experimental procedure (Pflügl-Haill et al., 2000). On the other hand also over-stabilization of the actin cytoskeleton in growing *Micrasterias* cells by jasplakinolide (Holzinger and Meindl, 1997) and chondramides (Holzinger and Lütz-Meindl, 2001) results in retardation of cell growth and in severe cell shape aberration in a similar way as induced by the actin depolymerizing agents (see above). In case of jasplakinolide the cells are filled with abnormal aggregations of actin filaments as shown by TEM, while chondramides induce abnormal F-actin lumps or dense F-actin batches in a time dependent manner as visualized by immuno fluorescence or phalloidin labeling. Additionally the internal structural organization of the *Micrasterias* cell is lost and organelles are displaced.

All these studies strongly indicate a regulatory role of the actin system in morphogenesis of *Micrasterias* (see also Lütz-Meindl and Menzel, 2000). Actin filaments are involved in transport of cell wall material containing vesicles to the plasma membrane that is achieved by cytoplasmic streaming. However, inhibition of cytoplasmic streaming induced by any impact on the polymerization status of the F-actin system cannot account for the formation cell shape aberrations, but would only result in retardation or inhibition of growth. The severe cell shape malformations that occur independently from the mode of the F-actin impairment indicate that a functioning F-actin dynamics is indispensable for morphogenesis in *Micrasterias*. These results also emphasize how important it is to have model systems in which growth and morphogenesis can be easily distinguished as it is the case in *Micrasterias*.

### Ionic Regulation and Signal Transduction

Early studies have revealed that a local influx of **calcium** at the growing lobe tips of *Micrasterias* accompanies cell differentiation (Meindl, 1982a). This is in good agreement with findings in tip growing higher plant cells such as pollen tubes or root hairs where the zone of calcium influx corresponds to the area of cell wall expansion (e.g., Hepler and Winship, 2010). During oscillating pollen tube growth stretch-activated calcium channels open and an intracellular tip-focused gradient of cytosolic calcium is established. In *Micrasterias* calcium influx is clearly correlated to cell pattern formation (Meindl, 1982a, 1985; Troxell and Scheffey, 1991; see also Meindl, 1993). When the first pair of indentations is formed, calcium influx can be found at four symmetrically arranged zones that develop to the four main lateral lobes during the subsequent growth step. As soon as these four lateral lobes, as well as the polar lobe that lags behind slightly, have reached a particular size, the calcium influx pattern splits again and the lobe tips become bifurcated. Due to relative slow growth velocity in comparison to, e.g., pollen tubes, it is not possible in *Micrasterias* to determine whether calcium influxes follow or precede cell wall growth.

Besides the occurrence of multipolar growth and the corresponding simultaneous calcium influxes at several sites, another difference in calcium regulation in *Micrasterias* is apparent when compared to tip growing higher plant cells. Ratio imaging by the calcium indicator fura-2 dextran revealed that no measurable intracellular calcium gradient is established during the outgrowth of the lobes of *Micrasterias* (Holzinger et al., 1995). This result was further corroborated by experimental perturbation of the intracellular calcium level by either injection of BAPTA-type buffers known to dissipate intracellular calcium gradients. As these experiments had no influence on cell growth and pattern formation in *Micrasterias* it was concluded that calcium is only important at the outermost growth zones in the area of the plasma membrane, possibly for fusion of the secretory vesicles. In contrast to tip growing higher plant cells such as pollen tubes (Holdaway-Clarke and Hepler, 2003) cytoplasmic calcium gradients do not seem to be involved in growth and cell shaping of *Micrasterias*.

That local calcium influx is correlated to cell pattern formation was demonstrated in a uniradiate variation of *M. thomasiana* (*Micrasterias thomasiana uniradiata*) in which the cell pattern is only expressed at one half of the cell. Only at the patterned side of the cell calcium influxes were measured or visualized by fluorescent markers, whereas at the non-patterned side no fluorescent signals were visible and outward directed currents were measured (Meindl, 1985; Troxell and Scheffey, 1991). Moreover experiments with ionophores, chelators or calcium channel blockers clearly showed a correlation between the intracellularly available calcium level and cell growth (for references see above and also McNally et al., 1983; Meindl, 1993). It was also demonstrated (Meindl, 1990) that formation of abnormal cell patterns as for example induced by elevated temperature is accompanied by a shift in the calcium distribution at the plasma membrane suggesting calcium influxes not at the lobe tips but at abnormally growing areas in between (see also below).

**Nitric oxide (NO)** which is regarded as key molecule for intracellular signaling and is involved in developmental and growth processes of higher plants but also in programmed cell death and defense mechanisms (see e.g., Murgia et al., 2004; An et al., 2005) has been shown to inhibit growth but not morphogenesis in *Micrasterias* (Lehner et al., 2009). The NO donors SNAP and SNP both arrest cell development, impaired dictyosomal function and prevented secondary wall formation. As the NO scavenger cPTIO (2-(4-carboxyphenyl)-4,4,5,5-tetramethylimidazoline-1-oxyl-3-oxide, potassium salt) abrogated SNP induced effects, it was concluded that growth inhibition was due to NO. It was hypothesized that NO inactivates enzymes of the secretory pathway such as GAPDH and thus provokes growth inhibition by preventing supply of cell wall material (Lehner et al., 2009). This indicates that a well-balanced level of NO is required for normal growth of *Micrasterias* cells.

**FIGURE 6:**
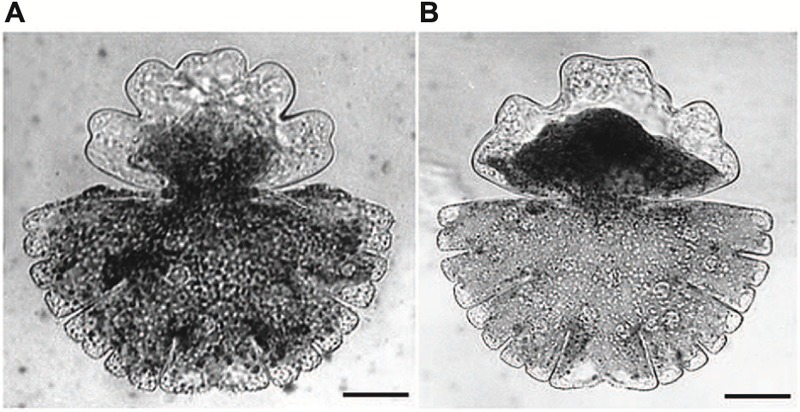
**Cell shape malformations of growing *Micrasterias* cells treated with the cholinergic antagonist D-tubacurarine **(A)** and with the agonist nicotine **(B)** during growth (scale bars are 30 μm).** Reprinted with permission from Schiechl et al. (2008), Copyright^©^ 2008 Elsevier Ireland Ltd.

**Acetylcholine**, one of the best investigated neurotransmitter in animal cells which has also been detected in moss, ferns and higher plants (Miura and Shih, 1984; Tretyn and Kendrick, 1991) has been also shown to be involved in growth and differentiation of *Micrasterias* (Schiechl et al., 2008). *Micrasterias* was the first unicellular alga in which a light-dependent production of acetylcholine has been proved by HPLC-coupled mass spectrometry. Both, cholinergic agonists (carbachol, nicotine) and antagonists (D-tubocurarine, hexamethonium) inhibited cell growth and evoked substantial cell shape aberrations when applied to early developmental stages (**Figures 6A,B**). Particularly the classic acetylcholine-receptor agonist nicotine additionally suppressed formation of the secondary cell wall. The presence of cholinergic receptors in *Micrasterias* was concluded from these results. They are obviously involved in growth of this alga and thus, like in higher plant cells, represent a basis for light induced differentiation (Schiechl et al., 2008).

To finalize this section it must be noted that our understanding of ionic regulation and signal transduction during of cell differentiation in *Micrasterias* is still in its infancy and that further studies will be required to obtain a more comprehensive insight.

## Stress Responses and Adaptation

### Temperature

Due to their worldwide distribution from tropical zones up to Polar Regions and high altitude mountains (Brook, 1981) desmids such as *Micrasterias* are generally well adapted to a wide range of water temperatures. On the other hand, even under moderate climatic conditions the small bog ponds the algae inhabit and that usually are not shaded by higher plants, can be exposed to rapidly changing seasonal and diurnal temperature conditions. The cells may face deep frost conditions in winter but also temperatures higher than 30°C during continuing heat periods in summer. While non-growing *Micrasterias* cells are better adapted, developing cells react highly sensitive to temperature changes with respect to growth and morphogenesis (Meindl, 1990; Weiss and Lütz-Meindl, 1999; Weiss et al., 1999).

Low temperature between 3 and 9°C generally retards growth and development in *M. denticulata* cells that were pre-cultivated at 20°C (Meindl, 1990). When exposed in early developmental stages shortly after mitosis, temperatures lower than 3°C inhibit growth of *Micrasterias* cells completely and lead to cell death within 4 h. A temperature range between 4° and 9°C allows growth, but within the 5 h required for normal differentiation at 20°C, only an undifferentiated bulb is formed. Primary wall growth under these low temperature conditions is continued up to 24 h and frequently cell shape malformations arise. They are mostly characterized by a simplification of the cell pattern, yet the basic symmetry of the cell is maintained. Cytoskeleton dependent processes like spreading of the chloroplast and migration of the nucleus back to its central position are retarded.

Temperatures up to 30°C only slightly accelerate the developmental process in *Micrasterias* but have no consequences on cell pattern formation (Meindl, 1990). When the cells are exposed to temperatures between 30 and 33°C the resulting size and cell pattern is clearly reduced. In a range between 33 and 36°C severe shape formations occur. Depending on the precise temperature and on the developmental stage at the beginning of treatment, the malformations are expressed in either a simplification of the cell pattern, or in formation of bizarre asymmetric cell shapes, or in development of multi-lobed cells with a higher number of lobes when compared to shapes formed at 20°C. The shape malformations go along with changes in patterned calcium distribution as indicated by a fluorescent marker (Meindl, 1990). Variations in cell size and pattern formation due to elevated temperature was also found in *M. rotata* indicating high temperature-related phenotypic plasticity (Neustupa et al., 2008).

In *M. denticulata* large areas of heat shock granules surrounded by ER cisternae are frequently found in the cytoplasm during elevated temperature (Meindl, 1990). The heat shock proteins hsp70 and BiP (binding protein) were detected by immuno-blotting. Hsp70 was found to be increasingly expressed after continuous or repeated experimental heat exposure (Weiss and Lütz-Meindl, 1999). Heat induced *de novo* synthesis of hsp70 reached its maximum after 9 h continuous heat exposure which corresponds well to findings in higher plant cells (see e.g., Ougham and Howarth, 1988). Within a certain temperature range (15 – 39°C), the intensity of the heat response in *Micrasterias* depends on the preceding cultivation temperature and on the duration of the heat exposure. Cells cultivated at 25°C generally react much weaker to heat than those grown at 15 or 20°C, indicating their distinct adaptive abilities (Weiss et al., 1999). Heat induced disturbance of cell shape formation is more pronounced after pre-cultivation at 15 and 20°C than at 25°C. In contrast, cell division rates are reduced more severely by heat after pre-cultivation at 25°C than after 20 or 15°C. Independent from the cultivation temperature, photosynthetic activity and respiration measured by polarographic oxygen production/consumption increase continuously and reach a peak in a range between 30 and 32°C. Thereafter photosynthesis drops to zero at 40°C after pre-cultivation at 15° but declines less distinctly after pre-cultivation at 20 or 25°C. These results show that the optimum temperature for photosynthesis in *Micrasterias* is similar to that determined in higher plants of temperate areas (Larcher, 2001) and that primary energy balancing processes such as photosynthesis and respiration are less affected by elevated temperature than cell division rates and cell shaping (Weiss et al., 1999). This fact may represent an important strategy for survival of the cells in their natural habitats. Good adaptation to different temperature regimes with respect to photosynthetic activity was also shown in closely related desmids collected at different climate zones (see for example Stamenkovic and Hanelt, 2013).

### UV Irradiation

*Micrasterias denticulata* cells show surprising high tolerance against experimental UV irradiation down to cut-off wavelength of 284 nm when exposed in the presence of white light in a sun simulator (Meindl and Lütz, 1996). Even in the sensitive stage of cell development, growth and pattern formation as well as cytoplasmic streaming are not affected by a 5 h treatment. When UV exposure is extended or the cut-off wavelength is lowered to 280 nm or 275 nm cell growth and differentiation are inhibited, cytoplasmic streaming is retarded, vacuoles are formed and the distribution of the large chloroplast that fills each semicell is disturbed. Drastic alterations of chloroplast structure ending up in more or less complete disintegration of grana and stroma thylakoids were observed (Lütz et al., 1997). These ultrastructural changes are reflected in a continuous decrease of photosystem II (PSII) activity as measured by fast chlorophyll fluorescence. The ratio between variable and maximum fluorescence (Fv/Fm ratio) reached very low levels around 0.1 after 1 h exposure to 280 nm or 275 nm UV cut-off wavelength, whereas untreated controls at culture conditions reached an averaged Fv/Fm value of 0.76. Photosynthetic oxygen production is maintained even at high UV irradiation with cut-off wavelengths of 275 nm for 15 min, but is completely suppressed upon prolonged treatment (Lütz et al., 1997).

In addition to the breakdown of chloroplast structure and photosynthesis the endomembrane system reacts distinctly to irradiation with low UV cut-off wavelengths. As a typical hallmark for stress (see below) the dictyosomes become involute, the number of their cisternae decreases and vesicle production is either reduced or completely inhibited (Meindl and Lütz, 1996). Abnormally large sheets of ER cisternae pervade the cytoplasm and microtubule re-polymerization during nuclear migration is prevented.

In summary these results show that like other desmids (see e.g., Holzinger and Lütz, 2006; Holzinger et al., 2009) *Micrasterias* is highly adapted to UV-B irradiation, which may explain the worldwide distribution and the presence of this group of algae even in high mountain areas or Polar Regions. It remains to be investigated how the considerable UV-B resistance of *Micrasterias* is achieved. As in other algae, a protecting function of the surrounding mucilage envelope or of the thick secondary cell wall, absorbance by particular metabolites and/or a very well developed physiological repair system may account for it (Oertel et al., 2004; Remias et al., 2012; Kitzing et al., 2014).

### Oxidative Stress

Unfavorable environmental conditions such as UV irradiation, high light intensities, drought, salinity or man-made entry of heavy metals by traffic or agricultural practices, as well as compounds like herbicides may cause oxidative stress in higher plants but also in aquatic photosynthetic organisms. Among the different reactive oxygen species (ROS) hydrogen peroxide (H_2_O_2_) represents a key molecule in biotic und abiotic stress and induces programmed cell death (PCD) in plant and animal cells.

Experimental application of H_2_O_2_ causes severe ultra-structural and physiological alterations in *M. denticulata* (Darehshouri et al., 2008). Swelling of mitochondria with simultaneous reduction of cristae, increase in the volume of ER as well as bending of dictyosomes associated with a bulging of their *cis*-cisternae and inhibition of vesicle production at the *trans*-side are the most pronounced structural effects of short-term H_2_O_2_ exposure. Photosynthetic activity measured by fast chlorophyll fluorescence decreased in these cells to Fv/Fm values of around 0.3 (see above). As the activity of caspase-like proteins known to be involved in PCD of plant cells (van Doorn and Woltering, 2005) increases in *Micrasterias* during H_2_O_2_ impact, it is likely that the cells undergo PCD. This is also corroborated by the fact that cell vitality is maintained, chromatin is slightly condensed and the increase in caspase-like activity is abrogated by a “classical” caspase-3-inhibitor (Darehshouri et al., 2008). Although molecular participants in PCD are not yet known these results clearly point toward the capability of *Micrasterias* to undergo PCD. This is in good agreement with studies on other unicellular algae such as *Dunaliella* (Segovia and Berges, 2005) or *Chlorella* (Zuppini et al., 2007) but represents the first report on PCD in a desmid. The physiological importance of PCD in *Micrasterias* may be explained by “altruistic cell death” (see also Lee et al., 2002). Under unfavorable environmental conditions PCD of a large number of cells may be beneficial for the survival of the population at their natural habitat. The surviving cells may use dead cells and the mucilage obtained from them for protection against further environmental impact (Darehshouri et al., 2008).

Oxidative stress following different kinetics of increased ROS production is also evoked in *Micrasterias* as consequence of experimentally elevated salinity, osmotic stress and by exposure to different heavy metal solutions (see below).

### High Salinity

In the small bog ponds that desmids like *Micrasterias* inhabit, the algae may face rapidly changing osmolarities of their surroundings water by evaporation during high temperature periods as well as by dilution during rain. Additionally the cells are endangered by increase in salinity which may occur due to agricultural practices such as fertilization or to road salt. In contrast to salt tolerant green algae such as *Dunaliella, Chlamydomonas* or *Chlorella* that have developed metabolic strategies to cope with elevated salinity (Pelah et al., 2004; Yoshida et al., 2004; Takagi et al., 2006; Goyal, 2007) particularly large-cell desmid such as *Micrasterias* usually growing only in rain-supplied acid bogs, are not at all adapted to increasing salt concentrations. This means that high salinity may represent a severe danger to the population.

In an early study (Meindl et al., 1989) it was demonstrated that the osmolarity of the nutrient solution markedly influences cell division rates of *M. denticulata.* An experimental increase in the osmolarity of the nutrient solution from the usual level (lower than 2 mosm/kg) up to 26 mosm/kg results in a steady decrease of the cell number as indicated by a particular cell division assay (Meindl et al., 1989). It ends up in a complete arrest of cell divisions at the highest osmolarity. *Micrasterias* cells that are not able to divide under these circumstances accumulate high amounts of starch grains, plastoglobules and oil bodies in their chloroplasts. Vacuoles of these cells appear highly electron dense in TEM indicating high salt accumulation. All together the appearance of these cells corresponds to storage stages that are also found at their natural habitats in winter or early spring (for further discussion and literature see Meindl et al., 1989). Interestingly cells kept at high osmolarity start dividing within 1 day when osmolarity is diminished in recovery experiments. This indicates that changes in osmolarity of the surrounding medium are important for switching off the cells from an inactive storage to an active division stage. The fact that during winter or other unfavorable environmental conditions such as drought, cell division is arrested, guarantees survival of the population by maintaining their energy balance. Dilution of the surrounding medium by rain immediately induces cell divisions.

**FIGURE 7:**
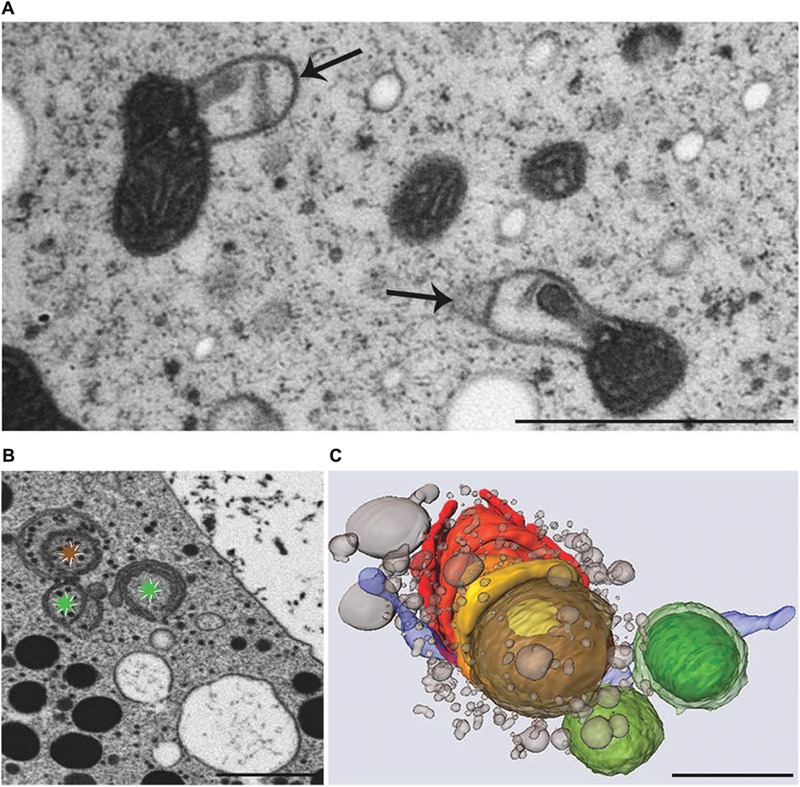
**Transmission electron microscopy micrographs of mitochondria **(A)**, FIB-SEM image of degrading dictyosome **(B)** and 3-D reconstruction of degrading dictyosome from FIB-SEM series **(C)** after exposure of *Micrasterias* to KCl. (A)** Mitochondria with balloon-like protrusions of outer membrane and condensed matrix. **(B)** The colored asterisks indicate parts of the dictyosome that are reconstructed in **(C)**. **(C)** The reconstruction shows that the dictyosomal cisternae form balls during degradation. Remnants of dictyosomal cisternae in red, ER in blue, small vesicles represent degradation products of cisternae. Scale bars are 1 μm. **(B,C)** Reprinted with permission from Lütz-Meindl et al. (2015), Copyright^©^ 2015 Royal Microscopical Society.

Experimental addition of KCl or NaCl to the culture medium leads to severe ultrastructural and physiological changes in *M. denticulata* that can be clearly distinguished from changes induced by osmotic stress (Affenzeller et al., 2009a,b). KCl (200 mM) caused the most pronounced effects by inducing foam-like vacuolization of the cytoplasm and severe morphological changes of mitochondria even after 3 h incubation. The outer membrane of all mitochondria showed balloon-like protrusions and their matrix appeared condensed indicating a kind of shrinkage induced by KCl induced intra-organelle osmotic changes (**Figure 7A**). Similar structural alterations of mitochondria are known from higher plant cells under anoxic conditions (Virolainen et al., 2002) and also from nerve cell during PCD (Muriel et al., 2000). Interestingly the severe structural changes did not influence their function in *Micrasterias*. Respiration in KCl exposed cells was not decreased (Affenzeller et al., 2009b).

**FIGURE 8:**
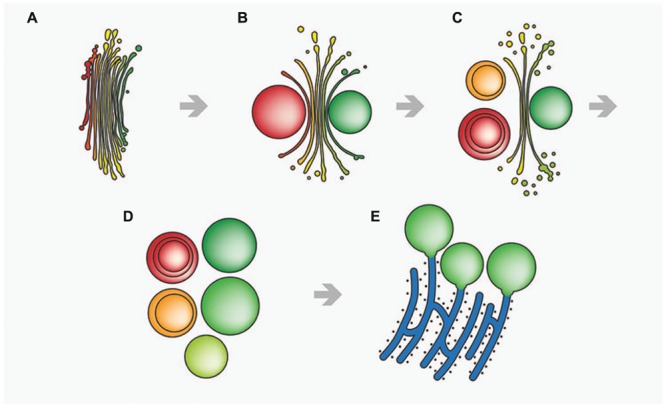
**Schematic drawing of stress-induced degradation of dictyosomes in *Micrasterias*. (A)** Control dictyosome with 11 cisternae (*cis*-side in red). **(B)** Beginning degradation, outermost *cis*- and *trans*-cisternae form balls. **(C)** Proceeding degradation and reduction of cisternal number. Cisternal balls include other cisternal balls. Numerous degradation products are visible close to the degrading dictyosome. **(D)** Dictyosome has completely disintegrated into balls. **(E)** The cisternal balls fuse with long, stress-induced ER cisternae. Reprinted with permission from Lütz-Meindl et al. (2015), Copyright^©^ 2015 Royal Microscopical Society.

In contrast, function of the endomembrane system was considerably impaired. ER cisternae were swollen and dramatically increased in number, whereas the cisternal number per Golgi stack decreased. Single dictyosomal cisternae were detached from the stack, both at the *cis*- and the *trans*-side. The remaining dictyosomal cisternae were completely inoperable as vesicles were no longer found in their proximity (Affenzeller et al., 2009b). When KCl treatment was continued for up to 24 h, degrading dictyosomes consisting of only two or three coiled cisternae were frequently found in contact with large ER compartments. 3-D analyses by FIB-SEM tomography provided evidence for the dictyosomal degradation process that occurred as consequence of KCl stress (**Figure 8**; Lütz-Meindl et al., 2015). Dictyosomal degradation starts with detachment of single cisternae from a stack. These cisternae form balls that may contain other cisternae of the same stack (**Figures 7B,C**). They increase in size by fusion with ER compartments and are finally absorbed by huge ER cisternae that pervade the cytoplasm. In contrast to our earlier assumptions (Affenzeller et al., 2009a,b) dictyosomes in *Micrasterias* are not disintegrated via autophagy. Although detailed studies on stress-induced dictyosomal disintegration are still missing there is also no evidence in the literature for autophagic degradation of dictyosomes in higher plant cells. Comprehensive articles that summarize selective autophagy in plants (Michaeli and Galili, 2014) report on autophagy of ER, mitochondria, plastids and peroxisomes, but not of dictyosomes.

Nevertheless structural hallmarks for autophagy do occur in salt stress *Micrasterias* cells. Both in NaCl and KCl exposed cells double membranes deriving from the ER partially surround or completely engulf peroxisomes indicating pexophagy (Affenzeller et al., 2009b). It seems that this autophagic process accompanies salt stress induced PCD. After both treatments cell vitality drops after 12 h exposure and is reduced to about 10% of controls after 48 h KCl incubation. DNA of NaCl and KCl treated *Micrasterias* cells fragments into discrete pieces of about 180 bp, a process referred to as DNA laddering and regarded as hallmark for PCD both in plant and animal cells (for discussion see Affenzeller et al., 2009a,b). DNA laddering occurs only after NaCl and KCl stress in *Micrasterias* but not after iso-osmotic sorbitol stress indicating that the ionic rather than the osmotic component of salt stress induces PCD. This is also reflected in the kinetics of ROS production. Like in higher plant cells (Zhu, 2001, 2002) salt stress in *Micrasterias* also causes enhanced ROS formation. Whereas the ROS level increases continuously within 3 h during osmotic sorbitol stress in *Micrasterias* it rises dramatically within 5 min during KCl and NaCl stress and then drops down to control level within 3 h (Affenzeller et al., 2009b). Because ROS are known as regulatory signals in cellular defense including PCD in higher plant cells (Lin et al., 2006) these different kinetics may be crucial for the induction of PCD. That cell death provoked by salt stress in *Micrasterias* is an active process is indicated by the fact that all structural alterations as well as DNA laddering occur at a point of time when metabolism is still unaffected. This is indicated by measurements of respiration, photosynthetic activity and plastid pigment composition.

By means of TEM-coupled EELS and statistical analyses, the chloroplast was determined as the main site of ROS, respectively H_2_O_2_ production in salt stress exposed *Micrasterias* cells (Darehshouri and Lütz-Meindl, 2010). Precipitation with cerium chloride also showed increased H_2_O_2_ levels in mitochondria, the cytoplasm and at the plasma membrane. The latter indicates an activation of the plasma membrane associated NADPH oxidase by salt stress, whereas the high values in the cytoplasm may result from ROS release by chloroplast and mitochondria (Darehshouri and Lütz-Meindl, 2010).

In summary, these studies show that imbalance of ionic homeostasis induced by salt stress, as in higher plant cells and yeast (Huh et al., 2002) triggers PCD in the theoretically “immortal” alga *Micrasterias*. By comparison to cell death events evoked by oxidative stress (see above) it becomes evident that one and the same organism is capable of performing different PCD pathways. This suggests that different stress inductors may activate different cellular reactions. An increase in caspase-3-like activity as found after oxidative stress in *Micrasterias* (Darehshouri et al., 2008) cannot be measured during salt stress (Affenzeller et al., 2009b). In contrast, DNA laddering accompanying cell death after salt stress does not take place during oxidative stress.

### Heavy Metal Impact

Increasing environmental pollution due to progressive traffic and industrial as well as agricultural production leads to the release of heavy metals into air, soil and water. Particularly aerosols generated by burning fossil fuels may enter ecosystems with high humidity such as peat bogs. It was demonstrated in several studies (e.g., Syrovetnik et al., 2004) that metals such as zinc, copper and cadmium may exceed the maximum permissible concentration in peat bogs by a factor of three or even higher. The low pH characteristic for peat bogs and ranging between 3.5 and 6.8 may increase the solubility of metals and thus enhances the threat to the ecosystem via accumulation of metals. Large-celled desmids such as *Micrasterias* growing exclusively under oligotrophic conditions are extremely sensitive to any contamination (Lenzenweger, 1996), particularly during their development.

Morphogenesis and cell development of *M. denticulata* are negatively affected by Zn, Al, Cd, Cr and Pb when applied at the highest concentrations that still allowed cell growth and decrease cell division rates in long-term experiments (Volland et al., 2011, 2012, 2014; Andosch et al., 2012). Correspondingly, photosynthetic activity measured by means of oxygen production and by fast chlorophyll fluorescence is reduced by almost all metals or completely abolished as in the case of CrVI. It reveals even negative values by application of low concentrated Cd (0.6 μM). Respiration in *Micrasterias* is markedly stimulated by every metal stress with highest values in Cu treated samples. This is not surprising as the repair and defense mechanisms induced by metal stress require high amounts of ATP. A similar increase in respiration during stress has been identified in *Micrasterias* during other stress scenarios (see above) and is also well known from stress response in higher plant cells (Larcher, 2001).

Plant cells and particular algae have different possibilities to cope with metal pollutants (see for example Rai and Gaur, 2001). In order to limit cytoplasmic metal concentrations they may attach metals to the extracellular matrix (cell wall and/or mucilage layer), actively excrete them into their surroundings or they may compartmentalize them in vacuoles or other storage compartments which frequently occurs by complexation by phytochelatines, metallothionines or strong chelators. To understand cellular metal detoxification and tolerance mechanisms it is thus important to determine intracellular targets of metals and to localize metals intracellularly. By means of TEM analyses and EELS (see above) that allows identification and semi-quantitive determination of element concentrations at highest possible spatial and energy resolution (see Lütz-Meindl, 2007) intracellular metal effects and sites of metal sequestration were determined in *M. denticulata.* It was shown that **aluminum** is only bound to the cell wall of *Micrasterias* when applied in long-term experiments. It was not found intracellularly but it induces abnormal depositions of primary material when applied during growth. This indicates that its binding to the cell wall matrix changes physical properties of the wall that account for the abnormal depositions and also for the shape malformations that are induced (for details and further discussion see Volland et al., 2011).

Similar results have been obtained after incubation of *Micrasterias* cells with **lead** that leads to severe cell shape malformation but is neither found in the cell wall nor in any intracellular compartment. It does not evoke any ultrastructural changes nor does it influence photosynthetic activity (Volland et al., 2014). It is therefore suggested as with Al, that Pb effects in *Micrasterias* are due to replacement of pectin-bound Ca^2+^ by the metal and that the arising changes in primary wall plasticity are responsible for the disturbed cell pattern formation. In fact Pb effects on cell shaping of *Micrasterias* could be almost completely abrogated by addition of Ca or Gd. The effects of inorganic lead in *Micrasterias* described above are in good agreement with findings of an earlier study where it was shown that PbCl_2_ caused bursting of the cells without any visible ultrastructural changes (Meindl and Röderer, 1990). In contrast, organic triethyl lead which was used as antiknock additive in fuels, evoked severe disturbance of the endomembrane system and of secondary cell wall formation.

High Ca^2+^-binding abilities of cell wall pectins have been shown for Al in *Micrasterias* (Volland et al., 2011) and have been suggested for Pb for example in moss (Krzeslowska et al., 2004). In this way the cell wall may act as a kind of filter by accumulating the metals thus preventing more severe intracellular damage. The result that Pb does not enter *Micrasterias* cells does not correspond to findings in higher plants, where Pb was frequently found to be taken up into the cytoplasm and to affect intracellular components (e.g., Jiang and Liu, 2010). It must be mentioned, however, that the considerable energy loss at the Pb M_4,5_ edge that was taken for identification of Pb by EELS and that induces unfavorable signal to noise ratios, may have prevented detection of low Pb amounts in *Micrasterias* cells (for discussion see Volland et al., 2014).

**Zinc** and **copper** were identified in cell wall precipitations of *Micrasterias* after long-term exposure (Volland et al., 2011). Additionally, both metals were found in mucilage vesicles which are secreted steadily in *Micrasterias.* Elimination of metals from the cytoplasm by using mucilage vesicles as fast vehicles seems to represents an important detoxification mechanism in *Micrasterias.* Increased mucilage production has been frequently observed in this alga under different stress scenarios. An increased number of structural interactions between mucilage vesicles and different cellular compartments are generally found in *Micrasterias* cells during stress. In lower number they are also visible in untreated high-pressure frozen *Micrasterias* cells in TEM (Aichinger and Lütz-Meindl, 2005) and have been interpreted as degradation and/or detoxification mechanisms for maintaining normal cellular turnover.

Zn is additionally compartmentalized in vacuoles of *Micrasterias* which become electron dense upon continuing Zn influence. Along with elevated oxygen levels in the same areas, a sequestration of Zn as oxide is indicated. A crucial role of the vacuole in metal detoxification has been frequently demonstrated in higher plants. In the metal-tolerant plants *Cardaminopsis* and *Armeria* (Neumann and zur Nieden, 2001; Heumann, 2002) as well as in *Zea* (Jiang et al., 2007) the vacuole was shown to be the main depot for Zn. In *Micrasterias* the relatively high tolerance to Zn concentrations up to 30 μM is probably due to the ability of the cell to compartmentalize the metal in the cell wall and in vacuoles and to excrete it via mucilage production (Volland et al., 2011). In its ability to survive relatively high Zn concentrations at least for a limited period of time *Micrasterias* corresponds to the Zn-tolerant green alga *Stigeoclonium* growing in polluted mining water and producing high amounts of phytochelatine-related peptides (Pawlik-Skowronska, 2003).

Among all metals investigated Cu is compartmentalized the best in *Micrasterias*. Besides sequestration in the cell wall and in mucilage vesicles Cu was also found as precipitates in starch grains where it may help to avoid toxic effects as long as starch is not catabolized (Volland et al., 2011). Long-term experiments revealed that non-growing *Micrasterias* cells can cope with low concentrated Cu for a limited period of time of 3 weeks as indicated by an only slight reduction of both photosynthetic efficiency and cell division rates. Nevertheless, as in other algae such as diatoms or the green algae *Chlorella* and *Chlamydomonas* (Nassiri et al., 1997; Mehta and Gaur, 1999; Franklin et al., 2000) Cu induces severe toxic effects in *Micrasterias* and leads to cell death in concentrations above 0.4 μM. The high redox potential of Cu ions may cause its extreme toxicity to algae that led to its use as algaecide.

**Chromium** widely used in industry and existing in two oxidative states has severe inhibitory effects on *M. denticulata* (Volland et al., 2012). Whereas cell development and pattern formation is almost completely suppressed by 1 mM solutions of both CrIII and CrVI, only CrVI evoked a complete arrest of cell divisions even when applied in the low concen-tration 5 μM for 3 weeks. Increased vacuolization, condensed cytoplasm, swollen mitochondria and involute dictyosomes were the main ultrastructural alterations after exposure of *Micrasterias* to CrVI. They are regarded as general stress hallmarks (see also salt- or other metal stress). Additionally and more specifically, electron dense precipitations in bag-like structures were found in random distribution along the inner side of the cell wall under CrVI impact. The latter structures contained Cr as measured by EELS along with elevated levels of iron and oxygen. As these Cr containing bags were located outside of the plasma membrane these results indicate that Cr is extruded from the *Micrasterias* cell in form of an iron-oxygen compound (Volland et al., 2012).

By means of atomic emission spectroscopy it was found that untreated *Micrasterias* control cells contain considerable amounts of about 1.3 g Fe/kg dry weight whereas the Cr content ranged around 5.8 mg/kg dry weight. When the cells are exposed to 10 μM CrVI, the Cr:Fe ratio shifted in favor of chromium from 1:200 to 1:1.5 after only 1 week. This implies that Cr in *Micrasterias* is taken up instead of Fe, which is also corroborated by the fact that divalent ions of Fe, Zn and Ca are able to diminish effects of Cr in *Micrasterias* (Volland et al., 2014). A similar competition between Cr, Fe, S and P for carrier uptake is also known from higher plant cells (Shanker et al., 2005) and Cr was shown to displace other metals and particularly Fe from reaction centers (Pandey and Sharma, 2003). In *Micrasterias* Cr uptake was decreased when the cells were treated with Fe prior to Cr exposure and Cr accumulations in “bags” were no longer measurable by EELS under these conditions. Cr also triggered rapid ROS formation in *Micrasterias.* Interestingly the induced ROS kinetics in *Micrasterias* followed a double peak, which is known to be indicative for oxidative burst known as defense mechanism in higher plants particularly as consequence of pathogen attack leading to PCD around the infection site (e.g., Mandal et al., 2011).

These investigations show that Cr in both oxidative states is readily taken up into the alga *Micrasterias* although Cr represents an unessential nutrient. Like in higher plant cells the highly water soluble anion CrVI is more toxic in *Micrasterias* than the cation CrIII. CrVI induces severe impact on ultrastructure and physiology of *Micrasterias* and interferes with Fe homeostasis.

**FIGURE 9:**
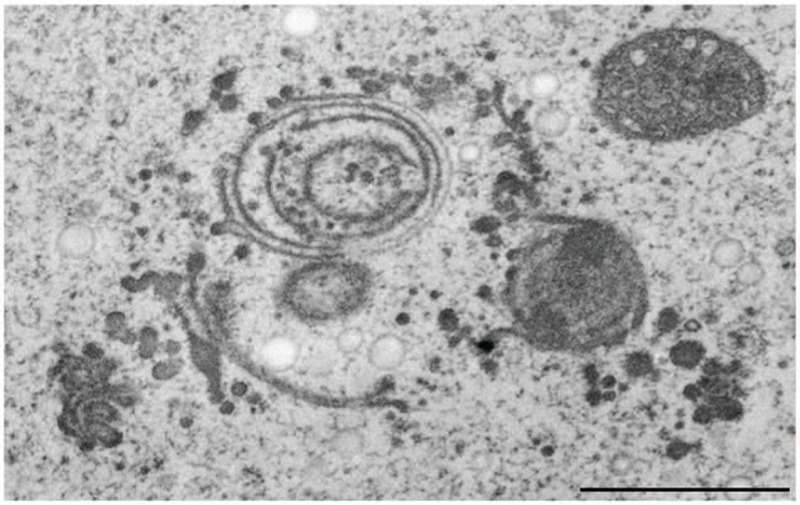
**Transmission electron microscopy micrograph of abnormal cluster of dictyosomes induced by treatment of *Micrasteria*s with CdSO_4_.** Scale bar 1 μm.

Among all metals tested on *Micrasterias* **cadmium** was the only one that, though extremely toxic to physiology and ultrastructure of the cells, was not compartmentalized intracellularly at all (Volland et al., 2011; Andosch et al., 2012). Cd is highly water soluble and enters aquatic ecosystems and soils mainly as consequence of anthropogenic activities such as disposal of electronic components. It represents a severe threat to human health by getting into the food chain via accumulation by plants (Templeton and Liu, 2010). Besides its severe negative implications on cell pattern formation, growth, photosynthetic activity and cell division (see above) it induces dictyosomal disintegration and autophagy in *Micrasterias* in concentrations of 150 μM (Andosch et al., 2012). Even a 1 h exposure to Cd resulted in a dramatic disturbance of ultrastructure, morphology and function of dictyosomes. The 11 cisternae of a control Golgi stack of *Micrasterias* were reduced to a maximum of 4 on the one hand, whereas on the other hand dictyosomal clusters that have completely lost their *cis-trans*-polarity (**Figure 9**) were found in Cd exposed cells. Vesicles were no longer found at the cisternal rims or in surrounding of the dictyosomes indicating a loss in secretion activity. The remaining inoperable dictyosomes consisted of only a few ring-shaped cisternae that were frequently surrounded by dilated ER cisternae (Andosch et al., 2012). Different stages of autophagosomes and autophagic vacuoles enclosing organelles, cytoplasmic portions or mucilage vesicles were present. Although cell viability was not affected, prolonged treatment with Cd for 1 day resulted in an almost complete depletion of dictyosomes in *Micrasterias*.

**FIGURE 10:**
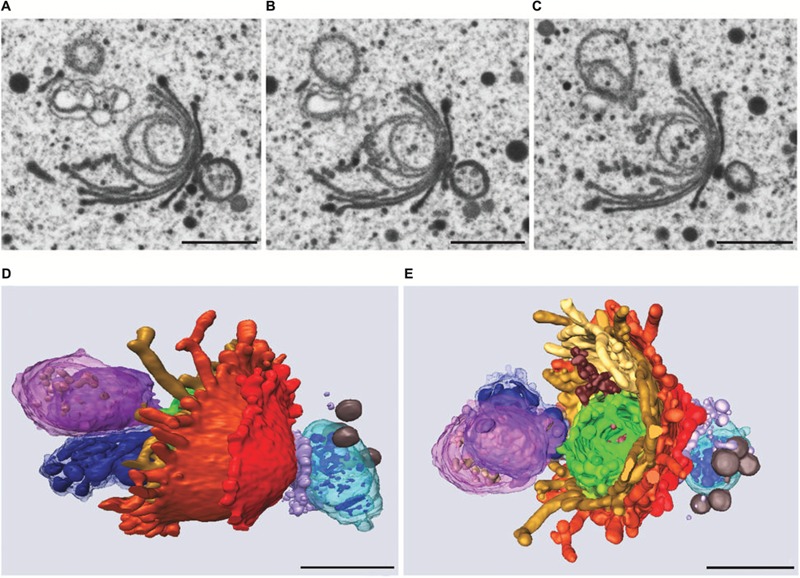
**Dictyosome of *Micrasterias* after exposure to CdSO_4_. (A–C)** Images from FIB-SEM series. **(D,E)** Different views of corresponding 3-D reconstruction. Dictyosomal cisternae from *cis-* to *trans-*side: light blue to violet. Cisternae form balls at *cis*- and *trans*-side, abnormal ER displayed in dark blue. Scale bars are 1 μm. Reprinted with permission from Lütz-Meindl et al. (2015), Copyright^©^ 2015 Royal Microscopical Society.

Similar to the results obtained during KCl stress, evidence was provided for a non-autophagic degradation of dictyosomes by means of FIB-SEM tomography also in Cd treated cells (Lütz-Meindl et al., 2015). **Figures 10A–E** show TEM images and a 3-D reconstruction of a dictyosome during beginning disintegration. Both at the *cis*- and the *trans*-side detached cisternae form balls whereas the remaining middle cisternae of a stack appear bent and their rims are markedly lacerated. This indicates that stress induced dictyosomal degradation follows the same way after salt stress and Cd exposure (see also **Figure 8**). Also the kinetics of ROS production during Cd exposure of *Micrasterias* corresponds to that during salt stress (Andosch et al., 2012). ROS production increases by a factor 7 within the first 30 min of Cd stress and drops down almost to control level after 4 days.

There are several indications that Cd exerts its negative effects on cell development, physiology and ultrastructure of *Micrasterias* by disturbing Ca homeostasis (Andosch et al., 2012). For example, Cd induced disturbance of structural and functional integrity of dictyosomes in *Micrasterias* can be mimicked by thapsigargin an inhibitor of plant and animal Ca^2+^-ATPase. This corresponds to results obtained in yeast where it was shown that Cd may affect Ca dependent Golgi ATPases (Lauer et al., 2008) that are also present in plant Golgi membranes (Ordenes et al., 2002). Abnormal cluster formation of dictyosomes during Cd exposure (see above) suggests a disturbing effect on the actin cytoskeleton that positions the Golgi bodies in desmids (see e.g., Url et al., 1993), via disturbance of Ca levels. Moreover addition of Ca to the culture medium prior to Cd exposure has been shown to prevent adverse Cd effects both on chloroplast structure and on photosynthetic activity in *Micrasterias* (Andosch et al., 2012). Also, the calcium channel blocker Gd was able to almost completely reverse negative impact of Cd on developing *Micrasterias* cells (Volland et al., 2014).

The ameliorating effects of Ca on Cd induced ultrastructural and physiological disturbances in *Micrasterias* are not surprising because of the ionic similarities between Ca and Cd. This implies that Cd may substitute Ca in essential metabolic pathways and may for example also bind to calmodulin. In case of its photosynthesis inhibiting effect, it obviously displaces Ca from the catalytic core of the photosystem II-water-oxidizing complex as shown in higher plants (Bartlett et al., 2008). Addition of Ca to the culture medium may then reactivate photosynthesis by preventing Cd uptake and binding (see also Volland et al., 2014). As similar ameliorating effects were observed after addition of Fe it was concluded that uptake of Cd is mediated in *Micrasterias* cells via both Ca and Fe plasma membrane channels (Volland et al., 2014).

By means of UPLC-mass spectrometry it was shown that Cd induces formation of phytochelatins in *Micrasterias* (Volland et al., 2013). Phytochelatins are known to be involved in detoxification of metals in higher plants (Ernst et al., 2008), fungi (Krauss et al., 2011) and green algae such as *Chlamydomonas* (Bräutigam et al., 2009) and others. Identification of PC_2_, PC_3_, and PC_4_ after 3 weeks exposure to Cd was the first proof of phytochelatins in an alga of the division Streptophyta (Volland et al., 2013). As the glutathione level of Cd treated cells was not significantly elevated it is assumed that glutathione required for phytochelatin biosynthesis is constantly supplied. It remains to be investigated in which way phytochelatins are involved in Cd detoxification in *Micrasterias.* Phytochelatines were neither detected in control cells nor in Cu exposed *Micrasterias* cells.

In summary these results show that physiological reactions of *Micrasterias* to different metals are similar, comprising decrease in photosynthetic activity and cell division rates, ROS production and increased respiration thus corresponding essentially to those of higher plants. At an ultrastructural level, chloroplast structure as well as morphology and activity of the endomembrane system seem to be the main metal targets. Filtering by the extraplasmatic matrix (mucilage and/or cell wall), excretion by mucilage vesicles and intracellular compartmentalization seem to be the most important detoxification strategies of *Micrasterias* that allow survival of the cells at least within certain concentration and duration limits.

## Conclusion and Outlook

This review shows that the alga *Micrasterias* is well suited as model system in plant cell biology for multiple reasons. The alga reveals an exceptional multipolar tip growth with several lobes developing simultaneously at the same plane. The continuously changing growing and non-growing zones of the primary cell wall allow for studying growth regulation in directly adjacent areas within one and the same cell and has high potential for providing insight into involvement of the cytoskeleton or ionic components in growth and cell shape formation. The alga develops two distinctly distinguishable cell walls that in their composition both resemble that of higher plant cells. The flexible highly pectic primary wall of *Micrasterias* is similar to cell walls in tip growing plant cells such as pollen tubes or root hairs and the protective thick cellulose-rich secondary wall corresponds to walls of non-growing higher plant parenchymatic cells. The extremely large dictyosomes that correspond to the large cell size provide excellent possibilities for investigating their function. They are enveloped by a huge ER sheath, consist of a constant number of 11 cisternae during the entire cell cycle and produce vesicle continuously even in non-growing periods. As most of their products are already well defined they lend themselves not only for basic cell biological studies on the function of the secretory machinery but represent also highly sensitive indicators for any kind of stress. Experimental or environmental impact on *Micrasterias* is easily recognizable not only by cell shape malformations but also by characteristic structural and functional reactions of the dictyosomes. They occur during high salinity or oxidative stress in the same way as during impact by different metal or herbicide pollutants indicating that dictyosomes in *Micrasterias* are highly sensitive stress sensors. Also degradation of dictyosomes under severe stress, achieved by detachment of single cisternae at both side of a Golgi stack occurs similarly during different stress scenarios. Degraded dictyosomes are absorbed by ER cisternae reflecting the high similarity between Golgi and ER membranes as also known from higher plant cells.

Despite their natural adaptation to oligotrophic, low salt-concentrated waters, *Micrasterias* cells are capable of coping with environmental pollution to a considerable degree. This is accomplished by an obvious physiological flexibility as well as by the capability of the *Micrasterias* cell of performing autophagy, and of compartmentalizing pollutants in cell walls and vacuoles, respectively, by excreting them via constant mucilage production. The molecular players that are involved in these processes as well as a possible regulation by signal molecules such as phytohomones remains to be investigated. Further establishment of molecular tools and sequencing of essential regulators combined with employment of high resolution 3-D microscopic techniques will enable the next steps for elucidating cell shape formation and for understanding intracellular stress response regulation. By its exceptional features and its close relation to higher land plants *Micrasterias* may help in detecting plant specific cellular processes and pathways that would remain undiscovered when only using “classical” model system such as *Arabidopsis*.

## Author Contributions

The author confirms being the sole contributor of this work and approved it for publication.

## Conflict of Interest Statement

The author declares that the research was conducted in the absence of any commercial or financial relationships that could be construed as a potential conflict of interest.
